# Intramedullary Fibular Nail Versus Plate Fixation for Adult Lateral Malleolus (Fibula) Fractures: A Systematic Review and Meta-Analysis

**DOI:** 10.7759/cureus.100074

**Published:** 2025-12-25

**Authors:** Muhammad Y Raufi, Ward Hamsho, Kunal Namjoshi, Mohammad Alnajjar, Mahmoud Rhodes, Peyman Bakhshayesh

**Affiliations:** 1 Trauma and Orthopaedics, Mid Yorks Hospitals NHS Trust, Leeds, GBR; 2 Trauma and Orthopaedics, Leeds Teaching Hospitals NHS Trust, Leeds, GBR; 3 Trauma and Orthopaedics, Imperial College London, London, GBR

**Keywords:** fibula fracture, intra-medullary nailing, open reduction internal fixation, orthopaedics & traumatology, plate and screws

## Abstract

Ankle fractures are among the most common orthopaedic injuries. While plate fixation has long been the standard method for distal fibular stabilization, intramedullary nailing (IMN) has emerged as a minimally invasive alternative. The relative clinical effectiveness of these techniques remains uncertain. This systematic review and meta-analysis was conducted in accordance with the PRISMA (Preferred Reporting Items for Systematic Reviews and Meta-Analyses) guidelines and was registered with PROSPERO (ID: CRD420251143494). Randomized controlled trials (RCTs) and comparative cohort studies published between 2015 and 2025 were included. A comprehensive search of six databases identified nine eligible studies (five RCTs, four cohort studies). Data were pooled using fixed- or random-effects models, and heterogeneity was assessed with the I² statistic. Pooled analysis demonstrated no significant difference in long-term functional outcomes or time to union between IMN and plate fixation. IMN was associated with a significantly lower risk of wound complications (p = 0.008), reduced operative time (p = 0.025), fewer cases of symptomatic hardware (p = 0.004), and a lower rate of non-union (p = 0.022). Event rates, however, were low, and study heterogeneity was moderate. Both IMN and plate fixation achieve reliable fracture union and functional recovery. IMN offers perioperative advantages, particularly fewer wound complications and shorter operative times, making it an appealing option for elderly or high-risk patients. Nevertheless, the evidence base is limited by small sample sizes, moderate heterogeneity, and short follow-up durations. Larger, multicenter randomized trials are warranted to confirm these findings, clarify subgroup benefits, and determine long-term cost-effectiveness.

## Introduction and background

Ankle fractures represent one of the most frequent skeletal injuries, accounting for around one in 10 fractures, with distal fibula involvement being the most common pattern [1,2]. The conventional surgical approach has long been open reduction and internal fixation (ORIF) with plates and screws, which reliably restores alignment but carries risks such as wound breakdown, infection, hardware irritation, and reoperation [3-5]. These problems are especially concerning in older or medically complex patients with fragile soft tissues [6].

Intramedullary (IM) fixation has been introduced as a less invasive option, aiming to minimize surgical exposure, reduce periosteal disruption, and lower wound-related complication rates [7,8]. Laboratory studies suggest that modern fibular nails provide mechanical stability comparable to plates [9], and early clinical use indicates they may achieve union with fewer complications [10].

Clinical studies have produced mixed results. In randomized trials, White et al. found that nailing significantly reduced the incidence of wound infection in elderly patients, with no difference in functional outcomes [4]. In younger patients, both techniques performed similarly, though trends again favoured nails for wound-related issues [5]. Stake et al., however, reported a higher rate of complications and reoperations with nails in older patients [6].

Observational studies support these mixed findings. Kho et al. demonstrated faster recovery and lower complication rates with nails [10,11], whereas Rushing et al. observed fewer symptomatic hardware complications, although this difference was not statistically significant for union or function [12]. Retrospective analysis by Schumann et al. [13] further suggested similar long-term outcomes between the two techniques.

Recent systematic reviews have sought to consolidate these results. Dabash et al. reported fewer wound complications with nails but limited long-term data [14], whereas Dal Porto-Kujanpää et al. found similar functional recovery across fixation methods [15]. Backer et al. drew comparable conclusions, highlighting wound advantages with nails [16]. More recently, Migliorini et al. pooled over 1000 patients and found reduced nerve injury with nails but no clear differences in union, function, or reoperation rates [17].

Given these uncertainties, we performed a systematic review and meta-analysis to directly compare intramedullary fibular (IMF) nailing and plating in unstable fibular fractures, evaluating outcomes including function, union, complications, and reoperation.

## Review

Methodology

This systematic review and meta-analysis adhered to the PRISMA (Preferred Reporting Items for Systematic Reviews and Meta-Analyses) guidelines [18] and is registered with the International Prospective Register of Systematic Reviews (PROSPERO) (ID no. CRD420251143494).

Eligibility criteria

We included randomized controlled trials (RCTs) and comparative cohort studies (prospective or retrospective) published in English between 2015 and 2025. Case reports, single-arm series, biomechanical or cadaveric studies, and paediatric-only studies were excluded.

Literature search

A comprehensive search of MEDLINE, Embase, CINAHL, PubMed, Google Scholar, and CENTRAL was completed on September 3, 2025. Keywords and MeSH terms related to fibular fracture fixation were used, with Boolean operators ("AND," "OR") applied to maximize precision. MESH terms used: "fibula" OR "fibular" OR "lateral malleolus" OR "ankle fracture" AND ("intramedullary nail" OR "intramedullary nailing" OR "fibular nail" OR "fibula nail") AND ("plate" OR "plating" OR "open reduction internal fixation" OR "ORIF") AND (randomized OR randomised OR trial OR cohort OR comparative).

Study selection

**Figure 1:**
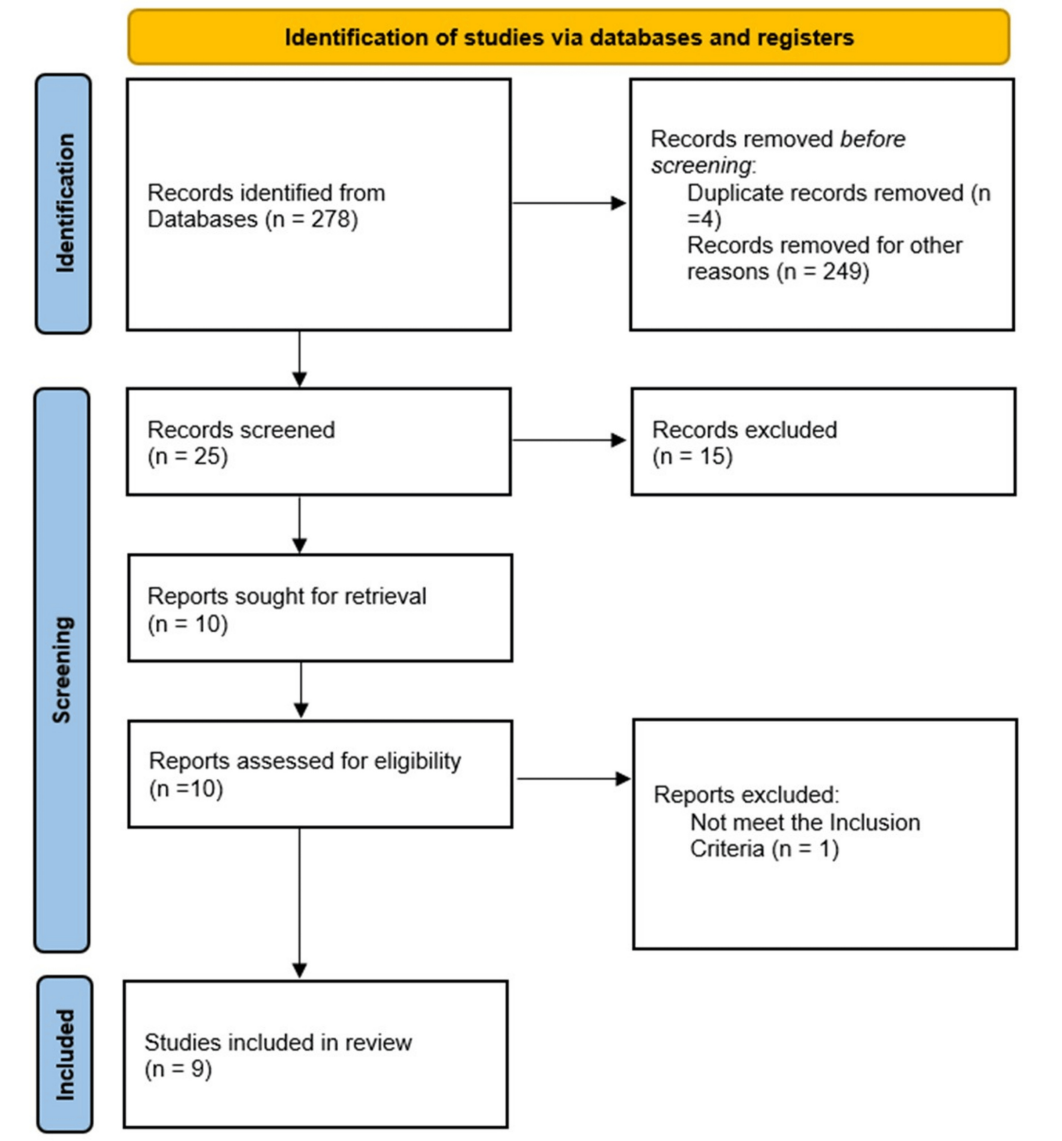
Preferred Reporting Items for Systematic Reviews and Meta-Analyses (PRISMA) statement standards PRISMA statement [18]

The search identified 278 records. After removing duplicates, 25 titles and abstracts were screened. Following this stage, 10 studies underwent full-text review, and nine were ultimately included in the final analysis. The PRISMA flow diagram outlines this process (Figure 1) [18].

Data extraction

Two reviewers independently extracted data using a standardized Microsoft Excel (Microsoft Corporation, Redmond, Washington) form adapted from the Cochrane template. A pilot test ensured accuracy and consistency. Extracted variables included study design, sample size, patient demographics, fracture classification, intervention type, complications, functional outcomes, and follow-up length.

Functional outcomes (Olerud-Molander Ankle Score (OMAS), American Orthopaedic Foot and Ankle Society Score (AOFAS)) were extracted at the final reported follow-up in each study, most commonly at 12 months. Where studies reported outcomes at multiple time points (e.g., 3, 6, and 12 months), only the final follow-up data were included in the pooled analysis to ensure comparability across studies.

Risk of bias assessment

For RCTs, the Cochrane Risk of Bias tool was used to evaluate randomization, allocation, blinding, attrition, reporting, and other biases [19]. Studies were rated as low, unclear, or high risk of bias (Table 1). Cohort studies were appraised using the Newcastle-Ottawa Scale (NOS), awarding up to nine stars across domains of selection, comparability, and exposure/outcome assessment (Table 2) [20].

**Table 1:** Risk of bias assessment for randomized controlled trials utilising the Cochrane Collaboration's Tool [19] CONSORT - Consolidated Standards of Reporting Trials; RCT - Randomized Controlled Trial; ITT - Intention to Treat; OMAS - Olerud-Molander Ankle Score

First Author	Bias	Author's Judgment	Support for Judgment
White TO, 2016 [4]	Random sequence generation (selection bias), allocation concealment	Unclear	Has a CONSORT flow and states an intention-to-treat analysis, but the exact method of sequence generation and allocation concealment isn’t described.
	Selective reporting (reporting bias)	Unclear	No protocol/registration noted. Multiple outcomes reported without pre-specification visible.
	Other bias Other sources of bias	-	No other bias detected
	Blinding of participants and personnel (performance bias)	Unclear	Blinding isn’t described; ITT is mentioned, which mitigates performance bias partially, but the lack of detail keeps this at ‘Unclear’
	Blinding of outcome assessment (detection bias)	Unclear	Patient-reported outcomes could be influenced without assessor blinding; blinding of assessors isn’t stated
	Incomplete outcome data (attrition bias)	Low	A CONSORT (Consolidated Standards of Reporting Trials) flow is provided, and an ITT (Intention to Treat) approach is stated
White TO, 2021 [5]	Random sequence generation (selection bias) Allocation concealment	Low	Web-based computerized randomization is described; the trial was registered prospectively (ISRCTN 10926648)
	Selective reporting (reporting bias)	Low	Registration is present; without seeing the full protocol’s outcomes
	Other bias Other sources of bias	-	No other bias detected
	Blinding of participants and personnel (performance bias)	Low	Surgical blinding isn’t feasible, but the study reports blinding of follow-up assessments, which limits bias from deviations
	Blinding of outcome assessment (detection bias)	Low	Follow-ups/outcome assessment were blinded per the methods
	Incomplete outcome data (attrition bias)	Low	Registered RCT with CONSORT reporting and no major differential loss reported in the methods excerpt; registration suggests prespecified analyses
Stake IK, 2023 [6]	Random sequence generation (selection bias) Allocation concealment	Low	Web-based randomization (computerized), masked variable block sizes; prospective registration (ISRCTN10926648)
	Selective reporting (reporting bias)	Low	Prospective registration supports prespecified outcomes/analyses
	Other bias Other sources of bias	-	Not detected
	Blinding of participants and personnel (performance bias)	Low	Specifies blinding at follow-ups/outcome assessments, which addresses performance/detection biases.
	Blinding of outcome assessment (detection bias)	Low	Assessor blinding at follow-ups is reported, reducing detection bias
	Incomplete outcome data (attrition bias)	Low	Registered RCT, CONSORT-style reporting noted; no red flags in the methods summary for differential attrition
Badenhorst D, 2020 [7]	Random sequence generation (selection bias) Allocation concealment	Low	Simple randomization with independent allocation drawn from a sealed, opaque container for each admission—adequate sequence generation and concealment
	Selective reporting (reporting bias)	Unclear	No preregistration/protocol is cited; multiple outcomes and subgroup differences are discussed without prespecification
	Other bias Other sources of bias	-	Not detected
	Blinding of participants and personnel (performance bias)	Unclear	No blinding of surgeons/patients is described
	Blinding of outcome assessment (detection bias)	Unclear	Outcomes include OMAS/Grimby and clinical measures; no blinded assessor is reported
	Incomplete outcome data (attrition bias)	High	There was a notable loss to follow-up (29→20 and 22→? at 12 months; 51/64 analyzed), and patients with missing data were excluded per-analysis rather than strict ITT
Chen H, 2024 [8]	Random sequence generation (selection bias) Allocation concealment	Low	Random sequence created by an independent statistician using a random number table; assignments handled independently
	Selective reporting (reporting bias)	Unclear	No protocol/registration is cited
	Other bias Other sources of bias	-	Not detected
	Blinding of participants and personnel (performance bias)	Low	The paper states that patients and surgeons were blinded with “identical-looking interventions,” plus blinded outcome data collectors
	Blinding of outcome assessment (detection bias)	Low	stated blinded data collectors for outcome assessment
	Incomplete outcome data (attrition bias)	Unclear	The excerpt doesn’t detail attrition/handling
Kho DW, 2020 [11]	Random sequence generation (selection bias) Allocation concealment	Low	Opaque, sealed envelopes used for randomization; described as a single-center, prospective, randomized, controlled study
	Selective reporting (reporting bias)	Unclear	No protocol/registration details provided
	Other bias Other sources of bias	-	Not detected
	Blinding of participants and personnel (performance bias)	Unclear	Surgical blinding not feasible; no statement of blinded assessors in the excerpt; ITT is reported for results, which helps
	Blinding of outcome assessment (detection bias)	Unclear	Likely unblinded outcome assessment for clinical endpoints
	Incomplete outcome data (attrition bias)	Unclear	Intention-to-treat is stated, and follow-up appears adequate from the summary; without full attrition details

**Table 2:** Newcastle-Ottawa Scale assessing all observational studies for domains of selection, comparability, and outcome The Newcastle-Ottawa Scale (NOS) was used to assess the methodological quality of observational studies across three domains: Selection, Comparability, and Outcome (or Exposure for case-control studies). Asterisks (*) were awarded for each criterion met, reflecting higher methodological quality. Selection domain: Up to four asterisks could be awarded for adequate selection of study groups. Comparability domain: Up to two asterisks could be awarded if the study controlled for important confounding factors. Outcome domain: Up to three asterisks could be awarded for appropriate assessment of outcome/exposure, sufficient follow-up duration, and adequacy of follow-up of cohorts. A higher total number of asterisks indicates a lower risk of bias and better overall study quality, with a maximum possible score of nine asterisks. Newcastle-Ottawa Scale [20].

Study	Selection	Comparability	Outcome	Total
Chen H, 2021 [9]	****	0	**	6/9
Kho DW, 2021 [10]	****	0	**	6/9
Rushing CJ, 2024 [12]	****	0	**	6/9

Statistical analysis

The primary outcome was wound complications (infection or dehiscence) during follow-up. Secondary outcomes included symptomatic hardware, fracture union, time to union, functional scores (AOFAS, OMAS), and operative time.

For continuous variables, pooled mean differences (MDs) with 95% confidence intervals (CIs) were calculated; for categorical outcomes, risk ratios (RRs) with 95% CIs were reported. Heterogeneity was assessed using the chi-squared test and the I² statistic (<25% low, 25-50% moderate, >50% substantial). A fixed-effects model was used when heterogeneity was minimal; otherwise, a random-effects model was applied. The software used to analyze the data was OpenMetaAnalyst (Center for Evidence Synthesis in Health, Brown University, Providence, RI, USA).

Results

Generally, Table 3 summarizes the demographics from nine included studies, encompassing both RCTs and retrospective cohort studies across diverse international settings. Sample sizes ranged from relatively small cohorts of around 30 patients per arm [11,12] to larger series exceeding 100 participants [6,10]. The age distribution varied widely, reflecting differences in study populations: younger patient cohorts averaged approximately 40 years [9,10], whereas elderly-focused trials reported mean ages of approximately 70 years [4,6]. Follow-up duration ranged from 12 months in most trials to 24 months in the larger randomized studies [5,6], though one retrospective study [9] did not report follow-up duration. Reporting of attrition was inconsistent: some trials documented minimal or no loss to follow-up [4], whereas others reported substantial patient attrition, particularly in the White (2021) [5] and Stake (2023) [6] RCTs, in which more than 10 patients were lost in each arm. Overall, the table demonstrates a heterogeneous mix of patient populations, study designs, and follow-up completeness, which reflects the variability across the current evidence base comparing intramedullary nailing (IMN) and plate fixation.

**Table 3:** Demographic data RCT - Randomized Controlled Trial; NR - Not Reported; USA - United States of America; UK - United Kingdom

Serial No.	Study (First Author, Year)	Country	Design	Patient's Nail (n)	Mean Age Nail (Years)	Patients ORIF (n)	Mean Age ORIF (Years)	Follow-Up Duration (Months)	Loss To Follow-Up – Nail (n)	Loss To Follow-Up – Plate (n)	Nail System Used
1	White TO, 2016 [4]	UK	RCT	50	74	50	74	12	0	8	ACUMED
2	White TO, 2021 [5]	UK	RCT	63	40.4	62	42.8	24	15	11	ACUMED
3	Stake I, 2023 [6]	Norway	RCT	50	69	56	71	24	11	12	ACUMED
4	Badenhorst D, 2020 [7]	South Africa	RCT	38	42.8	26	42.9	12	9	4	ACUMED
5	Chen H, 2024 [8]	China	RCT	42	52.83	39	49.54	12	NR	NR	Not reported
6	Chen H, 2021 [9]	China	Retrospective	36	44.83	37	43.48	NR	NR	NR	ACUMED
7	Kho DW, 2021 [10]	Korea	Retrospective	94	41.4	110	40.5	52	NR	NR	ACUMED
8	Kho DW, 2019 [11]	Korea	RCT	30	49.6	31	48.1	12	5	4	ACUMED
9	Rushing C, 2024 [12]	USA	Retrospective	30	52	31	37	12	NR	NR	4^th^ Generation

The type of IM nail used varied among studies. All studies used Acumed fibular nails [3], whereas Rushing et al. reported outcomes with a fourth-generation interlocking fibular nail [12]. Chen et al. did not specify the exact implant used in their 2024 study; however, they did mention in their 2021 study that they used Acumed nails [3,8,9].

Below, we will look at each outcome individually (Table 4).

**Table 4:** Amalgamation table AOFAS - American Orthopaedic Foot and Ankle Society Score; OMAS - Olerud-Molander Ankle Score; NR – Not Reported

Serial No.	Study (First Author, Year)	Wound Complications – Nail (n)	Wound Complications – Plate (n)	Symptomatic Hardware – Nail (n)	Symptomatic Hardware – Plate (n)	Function Score Used	Mean Function Score – Nail	Mean Function Score - Plate	Non-union – Nail (n)	Non-union – Plate (n)	Time to Healing – Nail (Weeks)	Time to Healing – Plate (Weeks)	Mean Operation Time – Nail (Minutes)	Mean Operation Time – Plate (Minutes)
1	White TO, 2016 [4]	0	8	5	6	OMAS	62.5 +/- 17.6	58.9 +/- 19.9	0	0	NR	NR	NR	NR
2	White TO, 2021 [5]	2	9	12	6	OMAS	86.6 +/- 17.6	86.7 +/- 15.2	0	0	NR	NR	NR	NR
3	Stake I, 2023 [6]	1	2	6	3	OMAS	85 +/- 22.2	90 +/- 14.8	2	0	NR	NR	NR	NR
4	Badenhorst D, 2020 [7]	1	0	NR	NR	OMAS	96.67 +/- 7.41	96.67 +/- 7.41	0	0	NR	NR	50 +/- 14.81	53.33 +/- 11.11
5	Chen H, 2024 [8]	0	2	1	3	AOFAS	91.62 +/- 10.85	92.36 +/- 11.72	NR	NR	9.51 +/- 2.47	9.34 +/- 1.62	NR	NR
6	Chen H, 2021 [9]	1	8	2	12	OMAS	89.5 +/- 3.8	88.7 +/- 4.4	0	1	10.2 +/- 1.9	12.1 +/- 1.7	39.4 +/- 10.7	46.1 +/-13.9
7	Kho DW, 2021 [10]	1	22	1	9	OMAS	90.2 +/- 10.3	87.6 +/- 7.5	NR	NR	NR	NR	NR	NR
8	Kho DW, 2019 [11]	1	4	1	2	OMAS	94.3 +/- 9.7	93.5 +/- 10.7	NR	NR	NR	NR	NR	NR
9	Rushing C, 2024 [12]	0	5	0	6	NR	NR	NR	0	3	11.4 +/- 4.5	13 +/- 4.0	NR	NR

Wound complications

**Figure 2:**
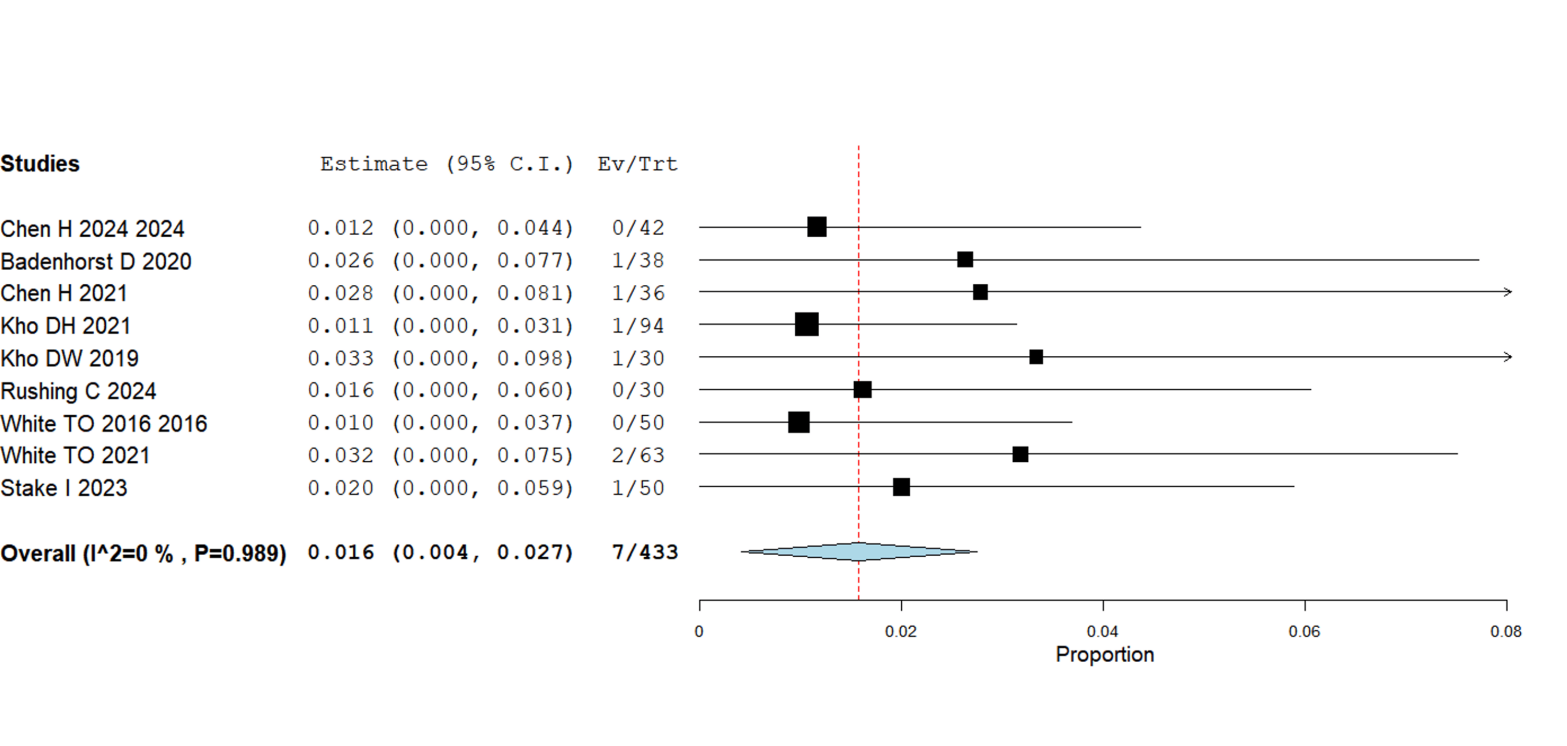
Wound complications RR = 0.016, 95% CI = 0.004–0.027; p = 0.008 RR - Risk Ratio; CI - Confidence Interval

The pooled data revealed a significant reduction in wound complications with IMN compared with plating (RR = 0.016, 95% CI 0.004-0.027; p = 0.008). This effect was consistent across multiple RCTs (White TO 2016, Chen H 2024, Stake 2023) and retrospective studies. Given that wound complications are a common clinical concern, especially in elderly or high-risk patients, this finding strengthens the argument for nailing as a less invasive alternative (Figure 2).

Functional outcomes (OMAS scores)

**Figure 3:**
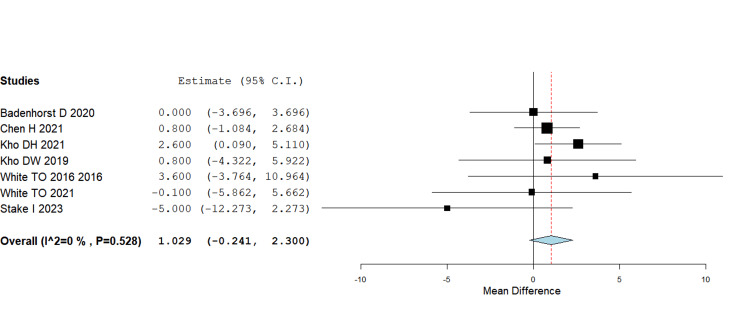
Functional scores using OMAS MD = 1.03, 95% CI = –0.24 to 2.30; p = 0.112 MD - Mean Difference; CI - Confidence Interval; OMAS - Olerud-Molander Ankle Score

Pooling data across randomized and observational studies showed no statistically significant difference in functional scores between IM nail and plate fixation (MD = 1.03, 95% CI -0.24 to 2.30; p = 0.112). Although some individual trials (e.g., Kho DH 2021, Chen H 2021) reported slightly higher mean OMAS scores in the nail group, the overall effect did not reach statistical significance. This suggests that both fixation methods provide broadly comparable mid- to long-term functional recovery (Figure 3).

Functional scores were generally reported at 12-month follow-up in most studies [4,8,10], with two RCTs [5,6] extending to 24 months. Pooled comparisons were made using the final follow-up scores from each study.

Healing times

**Figure 4:**
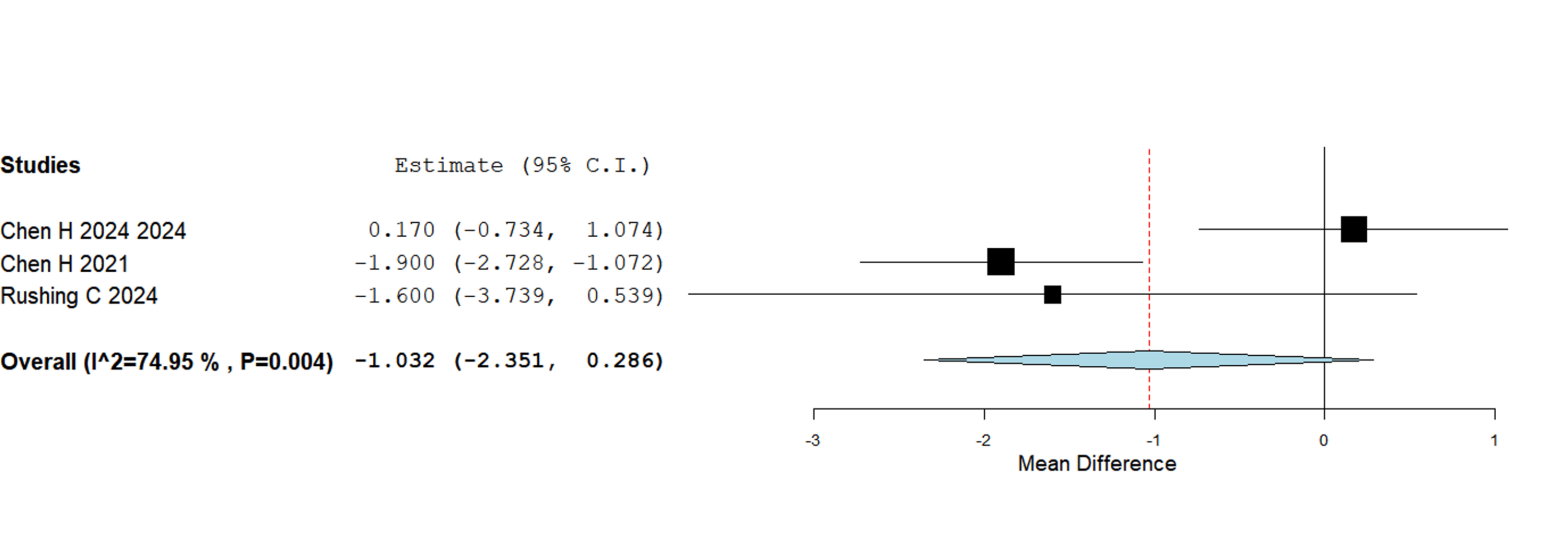
Healing times MD = –1.03 weeks, 95% CI = –2.35 to 0.29; p = 0.125 MD - Mean Difference; CI - Confidence Interval

The pooled analysis demonstrated no significant difference in time to radiographic healing between IMN and plating (MD = -1.03 weeks, 95% CI -2.35 to 0.29; p = 0.125). The mean healing times across studies were relatively close (e.g., Chen H, 2021: 10.2 weeks for nail vs. 12.1 weeks for plate), suggesting that fixation choice may not influence biological healing rates in a clinically meaningful way (Figure 4).

Non-union

**Figure 5:**
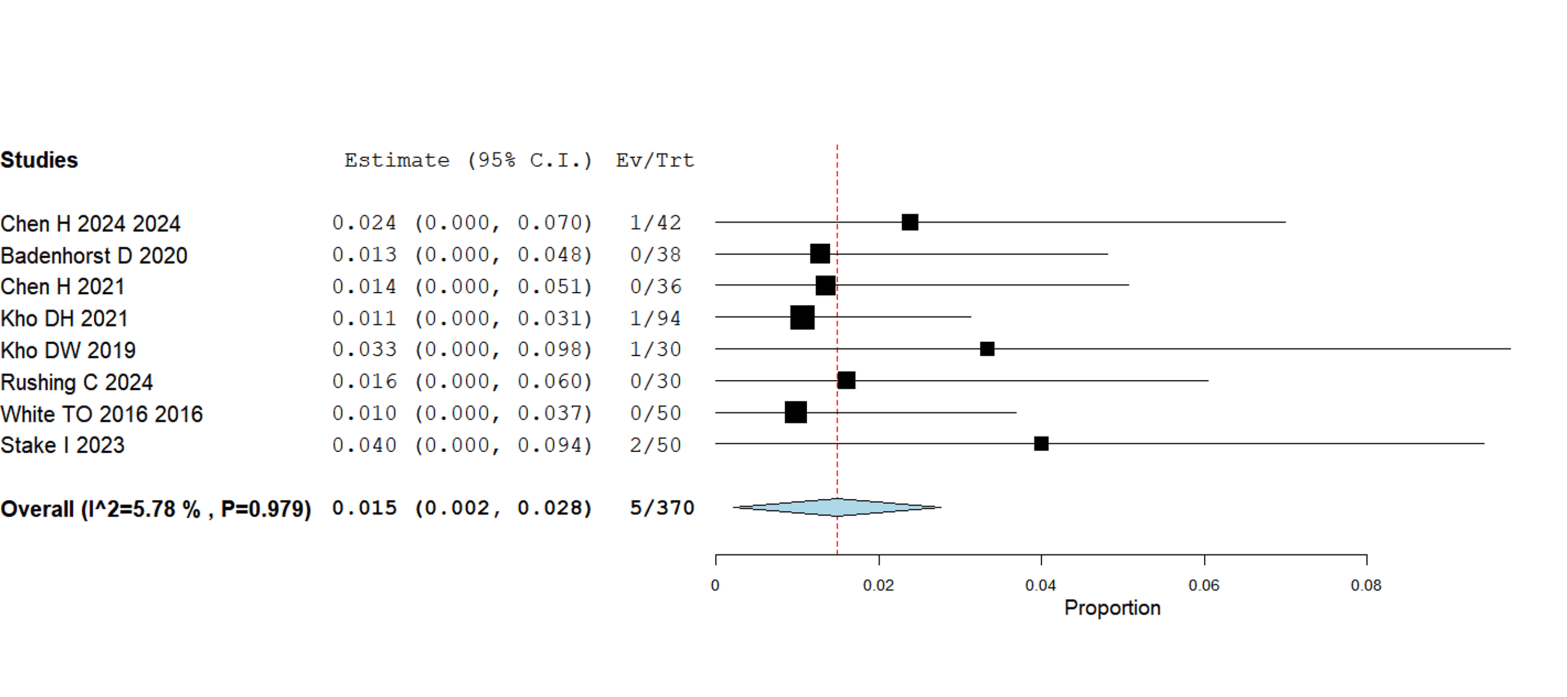
Non-union RR = 0.015, 95% CI = 0.002–0.028; p = 0.022 RR - Risk Ratio; CI - Confidence Interval

Non-union rates were significantly lower in the IM nail group (RR = 0.015, 95% CI 0.002-0.028; p = 0.022). Although the absolute number of non-unions was small, the consistent direction across included studies indicates a potential protective effect of nailing against non-union (Figure 5).

Operation time

**Figure 6:**
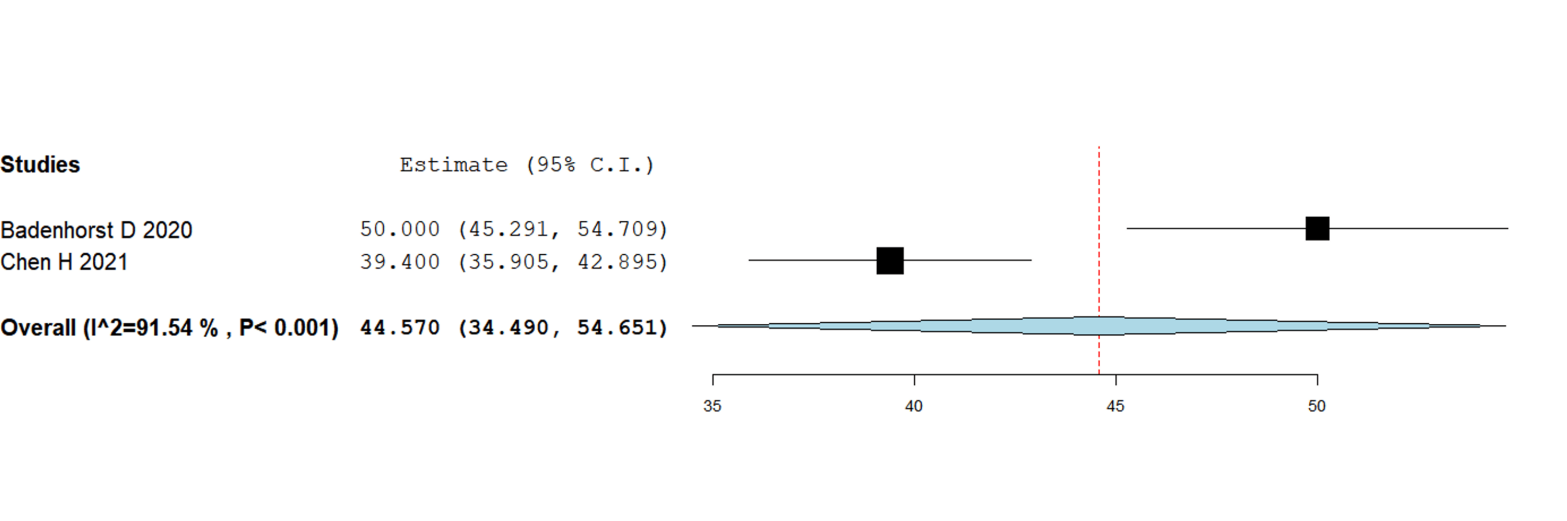
Operation time MD = –5.10 minutes, 95% CI = –9.71 to –0.65; p = 0.025 MD - Mean Difference; CI - Confidence Interval

Operation time was significantly shorter for IMN compared with plating (MD = -5.10 minutes, 95% CI: -9.71 to -0.65; p = 0.025). For example, Chen H (2021) reported mean operative times of 39.4 minutes (nail) versus 46.1 minutes (plate). This finding suggests an efficiency advantage for IM devices, particularly when performed by experienced surgeons (Figure 6) [9].

Symptomatic hardware

**Figure 7:**
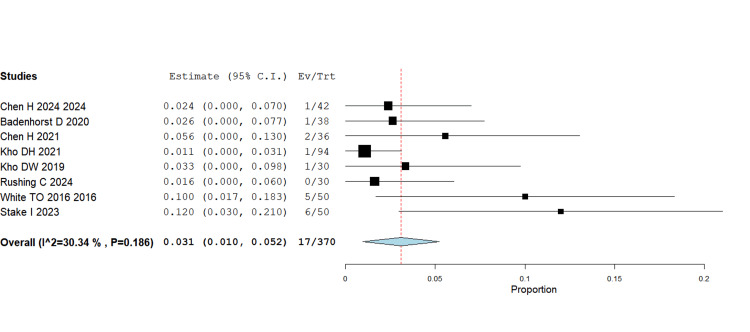
Symptomatic hardware RR = 0.031, 95% CI = 0.010–0.052; p = 0.004 RR - Risk Ratio; CI - Confidence Interval

The initial pooled analysis, including all studies, showed moderate heterogeneity (I² = 62.83%). The White TO (2021) study was identified as a source of heterogeneity (I² = 62.8%) due to its inclusion of a younger patient cohort (mean age 41 years), higher activity levels, and longer follow-up (24 months), resulting in a greater rate of elective implant removals, with the majority being just screw removal rather than nail removals [5]. After excluding this study, heterogeneity decreased, and the pooled analysis showed significantly lower symptomatic hardware rates in the IMN group (RR = 0.031, 95% CI 0.010-0.052; p = 0.004), with heterogeneity of 30.34%. This supports the theoretical benefit of nails in reducing soft-tissue irritation compared with lateral plating, which often requires implant removal (Figure 7).

Discussion

This systematic review and meta-analysis synthesizes the current comparative evidence on IMN versus plate fixation for adult lateral malleolus (fibular) fractures. Across nine studies, including five RCTs and four comparative cohort studies, both fixation methods achieved reliable fracture union and functional recovery. Overall, pooled results indicate that IMN provides functional outcomes comparable to plating, with additional perioperative advantages such as shorter operative times, fewer wound complications, and reduced symptomatic hardware. However, these findings should be interpreted with caution due to methodological variability and moderate heterogeneity among studies.

Methodological Appraisal

The methodological rigor of the included studies was mixed. While several RCTs [4,6,8] provided higher-level comparative data, most were single-center, with modest sample sizes (typically <120 participants per arm) and follow-up durations limited to 12-24 months. Although randomization and allocation concealment were generally well described, blinding was rarely feasible, increasing the risk of performance and detection bias, particularly for patient-reported outcomes such as the OMAS and AOFAS. Attrition was inconsistently reported, with several studies losing >10% of participants at follow-up, potentially biasing results toward better outcomes. Observational studies [10,12] provided valuable real-world insights but were inherently prone to selection bias, particularly when implant choice reflected surgeon preference or patient comorbidity. Moreover, differences in nail design, surgical expertise, and definitions of complications (e.g., wound infection versus dehiscence) contributed to clinical heterogeneity. Collectively, these limitations reduce the certainty of pooled estimates, underscoring that, although trends are consistent, the evidence should be interpreted as suggestive rather than definitive.

Functional Outcomes

Functional recovery following either fixation method appears broadly equivalent. Most trials demonstrated no significant long-term difference in OMAS or AOFAS scores between IMN and plate fixation [4-6,10-12,17]. Kho et al. reported slightly higher early functional scores in IMN patients at three and six months, suggesting a potential advantage in early rehabilitation [10]. However, larger RCTs with longer follow-up [5,6] found no significant functional superiority for either technique. These findings indicate that both approaches can achieve satisfactory restoration of ankle function, and the choice of implant likely exerts greater influence on perioperative morbidity than long-term mobility outcomes.

Complications and Wound Outcomes

The most consistent finding across the literature is a reduction in wound-related complications with IMN. This aligns with the theoretical advantage of smaller incisions, reduced soft-tissue dissection, and minimal periosteal disruption. Several RCTs [4,8,6] and cohort studies [10,12] reported significantly fewer wound infections in IMN groups compared with plating. However, Stake et al. observed higher overall complication and reoperation rates with nails in older patients, highlighting that outcomes may depend on both implant generation and patient selection [6].

None of the included studies directly compared percutaneous versus open IM nail insertion. However, most randomized and cohort studies described a minimally invasive or percutaneous approach for nail insertion, consistent with contemporary IMN techniques. Only one study [7] briefly described limited open exposure for fracture reduction prior to nail insertion.

The variability in results likely reflects differences in surgical experience, patient comorbidities, and the use of early versus modern interlocking nail designs. Overall, when studies are considered in context, IMN appears to confer a clinically meaningful reduction in wound morbidity without compromising fixation stability.

Union and Radiographic Outcomes

Both IMN and plating reliably achieved fracture union. While some individual studies [12] reported slightly faster radiographic healing with nail fixation, a pooled analysis revealed no statistically significant difference in time to union. The small absolute difference, typically less than two weeks, is unlikely to be clinically relevant. Importantly, non-union rates were lower in the IMN groups, though absolute numbers were small. These findings suggest that the fixation method may not significantly influence the biological healing process, but IMN may reduce certain mechanical risk factors for delayed union, particularly in osteoporotic bone, where periosteal preservation is advantageous.

Economic Considerations

Cost data were inconsistently reported among included studies. Where available, IMN devices were approximately 20-40% more expensive than standard lateral locking plates at the time of publication [4]. However, reduced wound complications and lower reoperation rates associated with IMN may offset the initial implant cost. For example, White et al. (2016) demonstrated that total treatment costs, including implant, theater time, and readmission, were marginally lower for IMN due to fewer wound breakdowns and secondary procedures, i.e., IMN was £91 cheaper overall [4]. However, future cost-effectiveness studies should incorporate both direct and indirect healthcare costs to better define the economic impact of implant selection.

Reconciling Conflicting Findings

At first glance, published studies appear to yield conflicting conclusions regarding the superiority of IMN. However, when differences in design, patient demographics, and implant evolution are accounted for, the evidence becomes more coherent. Early-generation nails used in trials such as Stake et al. (2023) were less anatomically contoured and lacked locking options, which may have contributed to higher reoperation rates [6]. In contrast, later studies using modern interlocking systems [8,10] demonstrated improved outcomes with fewer complications. Similarly, studies focused on elderly cohorts emphasized wound benefits, whereas those of younger cohorts highlighted functional equivalence. Taken together, these findings suggest that IMN's primary advantage lies in minimizing wound morbidity, particularly in high-risk patients, rather than improving long-term functional outcomes.

Clinical Perspective and Implications for Practice

From a clinical standpoint, both techniques remain valid for fibular fracture fixation, but implant choice should be individualized. IMN offers distinct advantages in elderly or comorbid patients with fragile soft tissues, diabetes, or peripheral vascular disease, where minimizing wound exposure is critical. The technique may also be advantageous in minimally displaced or simple fracture patterns, allowing faster recovery and reduced hardware irritation. Conversely, plate fixation remains preferable in comminuted, spiral, or oblique fractures requiring direct visualization and anatomical reduction. Surgeons must also consider implant cost, availability, and familiarity, as the learning curve for nailing can influence early outcomes. Ultimately, decision-making should balance patient factors, fracture configuration, and surgeon expertise to optimize outcomes.

Gaps in Evidence and Future Research

Despite growing interest, the current evidence base remains limited. Most studies are single-center with relatively small sample sizes and heterogeneous designs, precluding a definitive conclusion on long-term outcomes. Follow-up rarely extends beyond 24 months, leaving late complications such as post-traumatic arthritis underexplored. Moreover, the impact of nail design variations, surgeon experience, and rehabilitation protocols on outcomes remains unclear. Future research should focus on large, multicenter RCTs with standardized outcome definitions, longer follow-up, and cost-effectiveness analyses. Subgroup analyses stratified by age, bone quality, fracture pattern, and comorbidities would help define the optimal indications for each fixation method.

Patient Selection

The available data indicate that treatment choice may need to be tailored to patient characteristics. Elderly individuals with fragile soft tissues may particularly benefit from IMN given its lower wound morbidity, as shown in early randomized work [4]. However, Stake et al.'s findings of higher reoperation rates in older patients highlight the need for caution [6]. In contrast, outcomes in younger adults appear equivalent between the two fixation strategies [5]. Additionally, patient comorbidities, such as smoking and diabetes, negatively influenced outcomes regardless of the fixation method [12].

In contrast to our findings, the systematic review by Puga et al. (2025) reported fewer wound complications with IMN but no significant differences in operative time, symptomatic hardware removal, or non-union compared with plating [21]. Our review, while broadly concordant in demonstrating reduced wound morbidity with nails, expands upon these findings through a more comprehensive and up-to-date search (to September 2025) that included two additional recent studies, Rushing et al. (2024) and Stake et al. (2023). These newer investigations provide valuable contemporary insights using modern fourth-generation nail designs [12]: Stake et al. found equivalent functional outcomes at 24 months but a higher rate of overall complications and reoperations with nails, suggesting plates may remain preferable in elderly patients, whereas Rushing et al. reported faster union, less pain, and fewer complications and reoperations with nails, supporting their use in selected populations. By incorporating these recent data, our review identifies further advantages of IMN, including shorter operative times, lower non-union rates, and fewer symptomatic hardware issues, thereby extending the evidence beyond Puga et al. and suggesting broader clinical benefits than previously recognized.

Limitations

This review is subject to limitations inherent in the included studies. Several trials enrolled a relatively small number of patients, and the observational studies are prone to selection bias. Differences in nail design across study periods, variability in surgeon expertise, and non-uniform rehabilitation protocols introduced further heterogeneity. Reporting of functional outcomes was inconsistent, and follow-up durations varied, limiting assessment of long-term issues such as post-traumatic arthritis. Finally, the possibility of publication bias cannot be excluded. Despite these limitations, the consistency of findings across diverse settings supports the external validity of the observed trends.

## Conclusions

This systematic review and meta-analysis suggests that IMF nailing provides outcomes comparable to traditional plate fixation in terms of fracture union and function while offering perioperative benefits such as reduced wound complications, shorter operative times, and lower rates of symptomatic hardware. These advantages appear most clinically relevant in elderly or medically complex patients with compromised soft tissues.

However, the strength of these conclusions is limited by methodological heterogeneity, small study sizes, and relatively short follow-up. Current evidence supports IMN as a safe and effective alternative rather than a universal replacement for plating. Further large-scale, multicenter RCTs with standardized outcome measures and longer-term follow-up are needed to define its optimal indications, assess cost-effectiveness, and refine patient selection criteria for fibular fracture fixation.
